# Use of a trauma registry to drive improvement in the regional trauma network systems in Hawassa, Ethiopia

**DOI:** 10.1007/s00590-022-03410-z

**Published:** 2022-10-29

**Authors:** Mengistu G. Mengesha, Clara Vella, Ephrem G. Adem, Sintayehu Bussa, Lewam Mebrahtu, Andualem Y. Tigneh, Claude Martin, W. J. Harrison

**Affiliations:** 1 Countess of Chester NHS Foundation Trust, Chester, UK; 2 Hawassa University Comprehensive Specialized Hospital, Hawassa, Ethiopia; 3 AO Alliance, Davos, Switzerland

**Keywords:** Trauma registry, Ethiopia, Low- and middle-income countries (LMIC’s), Open fracture, Trauma

## Abstract

**Aim:**

Our aim is to establish and analyse the first year of trauma registry data from Hawassa University Comprehensive Specialised Hospital (HUCSH)—an Ethiopian tertiary referral centre. We plan to identify possible trends in injury patterns, access to health care and referral pathways and establish if our observations are in keeping with data published from other sub-Saharan LMIC’s.

**Methods:**

Prospective data collection using the WHO trauma registry dataset. All trauma patients presenting to HUCSH between November 2019 and November 2020 were included. Military patients were excluded. Dataset: Age, sex, region of residence, mode of transport to hospital, referral centre, time from injury to arrival in HUCSH, arrival triage category, Kampala Trauma Score (KTS), mechanism of injury, injury type, closed/open fracture and 24 h outcomes. Data statistical analysis was performed to calculate frequencies of the above variables.

**Results:**

There were a total of 1919 cases. Fifty-three per cent were caused by road traffic collision and 49% were fracture/dislocations. Public transport was the most common mode to hospital—40%. Seventy-seven per cent of all trauma admissions were referred from other centres, 69% had a mild KTS. A total of 376 patients presented with an open fracture—76% had a low KTS and 67% remained in ED for > 24 h. Sixty-five per cent of ambulances were utilised for mild KTS patients, only 25% of ambulances transported moderate and severe injuries.

**Conclusion:**

We have shown that a ‘one size fits all approach’ should not be adopted for LMIC’s as trends vary between regions and countries. Improvements are needed in ambulance utilisation, the use of appropriate triaging tools to facilitate initial basic trauma care and appropriate, timely referrals and the management of open fractures.

**Supplementary Information:**

The online version contains supplementary material available at 10.1007/s00590-022-03410-z.

## Introduction

Injuries account for 10% of deaths worldwide each year, 1.7 times more than deaths caused by HIV, Malaria and Tuberculosis combined [1]. Beyond this, it is estimated that, every year another 973 million people sustain injuries that will require some form of health care [2]. The burden of injury across the world is disproportionate—with 90% of injury-related deaths occurring in low- and middle-income countries (LMIC’s) [1]. Given injury morbidity and mortality is known to significantly reduce with improvements in the prevention and care of trauma patients [3], we have seen significant advances in the development and utilisation of trauma care systems in high-income countries (HIC’s). These registries provide valuable data for injury surveillance, health system development and resource allocation. Even though the burden of such injuries is higher in LMIC’s, the adoption and implementation of these registries has lagged behind [4].

Ethiopia is a landlocked country, sharing its borders with Somalia, Kenya, South Sudan, Sudan, Eritrea and Djibouti. With a population of over 112 million, Ethiopia is the second most populous nation in Africa, as well as one of the poorest per capita [5]. Devastating military confrontation in the northern Tigray region of the country has displaced over one million people, exacerbating pre-existing problems with access to health care and resulting in regional health system collapse [6]. Characterizing the burden of trauma on a local scale in surrounding regions is vital for improving resource allocation and preparedness in hospitals [7].

The aim of our study is to establish and analyse the first year of trauma registry data from Hawassa University Comprehensive Specialised Hospital (HUCSH)—a tertiary referral centre. Hawassa is one of the largest cities in southern Ethiopia, with a population of approximately 319, 023 [8]. HUCSH is located just over 250 km from the country’s capital city of Addis Ababa and is one of the busiest tertiary referral centres in the country. It has a catchment area covering four provinces—serving a population of more than 20 million people. Health care in Ethiopia is organised into a three-tiered system. Primary care is made up of health posts and primary hospitals; secondary care services are carried out by general hospitals and tertiary care by specialised hospitals [9].

This paper is both topical and pertinent as we focus on a region which has felt the effects of the conflict in Tigray, despite being over 1000 km away. This comes on a background of high volumes of trauma relating to road traffic collisions (RTCs). In the not-too-distant future, HUCSH may have to deal with a significant increase in trauma volume, late presentations (secondary to worsening access to health care) and potentially, a higher proportion of more severe injuries. The ability to collect, analyse and interpret reliable and relevant data is imperative to developing an evidence-based, resilient and efficient trauma care and referral system. We plan to identify possible trends in injury patterns, access to health care and referral pathways in trauma patients. We hope to establish if our observations are in keeping with trauma registry data published from other sub-Saharan LMIC’s—dispelling or reinforcing a ‘one size fits all’ approach. We hope this study will aid future development of existing trauma care systems and injury prevention framework in Hawassa and beyond.

## Methods and materials

Ethical approval was granted by the Institutional Review Board (IRB) of Hawassa University, College of Medicine and Health Science, registration number IRB/276/12, on the 3rd of December 2019.

Data were collected prospectively using the WHO trauma registry dataset. All trauma patients presenting (directly or referred) to HUCSH over a 12-month period—between November 2019 and November 2020—were included. Due to the COVID-19 pandemic, data collection was temporarily suspended for 2 months between—23 March 2020 and 25 May 2020.

Patients were tracked by trained data clerks, for a period of 24 h—from their time of arrival in the Emergency Department. Data were collected by trained healthcare professionals—emergency nurses, interns and residents—using the web-based WHO data collection tool. A backup, paper copy for each patient was stored securely in the hospital research office.

The following dataset was collected: Age, sex, region of residence, mode of transport to hospital, referral centre, time from injury to arrival in HUCSH (hours), triage category on arrival to HUCSH, Kampala Trauma Score (KTS), mechanism of injury, injury type, closed vs open fracture and 24 h outcomes. Trauma patients were defined; military patients were excluded from the study.

Injury severity was categorised as per the Kampala Trauma System [10] into mild, moderate and severe (Table 1). All in-hospital deaths have been included, any deaths outside of HUCSH have been excluded.

**Table 1:** Kampala Trauma Score

Component	Score
*Age (years)*	
5–55	2
< 5 or > 55	1
*Systolic blood pressure (mmHg)*	
> 89	4
50–89	3
1–49	2
Undetectable	1
*Respiratory rate (/min)*	
10–29	3
> 30	2
< 9	1
*Neurological status*	
Alert	4
Responds to verbal stimuli	3
Responds to painful stimuli	2
Unresponsive	1
*Serious Injuries*	
None	3
1	2
≥ 2	1
*Total Score*	
Mild	14–16
Moderate	11–13
Severe	< 11

Data analysis was carried out using Microsoft Excel, and descriptive statistics were done to calculate frequencies of the variables described above.

The HUCSH trauma registry project was supported financially by the AO Alliance, a developmental organisation committed to improving trauma care in LMICs [11].

## Results

Over the 12 month period, there were a total of 1919 recorded trauma cases. The majority of patients were male (80%) with an average age of 28.5 years, ranging from < 1 year to 112 years.

The most common mechanism of injury was road traffic collision (RTC), which accounted for 53% of all cases. This was followed by falls (18%) and blunt force trauma (10%). Assaults accounted for 7% of cases; gunshot wounds 3%, burns 1% and ‘other injuries’ 7%.

The most common mode of transportation to the hospital was public transport (40%); this was followed by ambulance (39%), private vehicle (20%) and other (1%). Ninety per cent of ambulance usage was to transfer patients from other healthcare centres (Table 2).

**Table 2:** Ambulance utilisation for referred trauma patients vs KTS

Kampala Trauma Score (KTS) versus mode of transport	Total referred patients
*Mild KTS*	*1016*
Ambulance	425 (42%)
Public transport	408 (40%)
Private transport	180 (18%)
*Moderate KTS*	*247*
Ambulance	161 (65%)
Public transport	51 (21%)
Private transport	35 (14%)
*Severe KTS*	*4*
Ambulance	3 (75%)
Public transport	1 (25%)
Private transport	0

The most common injury type was fracture or dislocation (49%), followed by head injury which was the primary injury in 37% of patients.

There were a total of 26 (1.4%) recorded deaths, 77% of these were referred patients. Sixty-five per cent were moderately graded injuries, according to the KTS. Head injuries accounted for 69% of all deaths; there was one death associated with an open fracture/dislocation.

With regard to referral source, 77% of all trauma admissions were referred from other centres. Eighty-three per cent of referred patients came from district general hospitals, 11% from health centres and 6% from private hospitals. The majority of referred patients had a mild KTS (69%); only 17% of patients referred had a moderate KTS and < 1% had severe KTS. We were unable to calculate the KTS for 14% of patients due to the absence of blood pressure documentation/monitoring.

Regarding ‘time to hospital’, the more severe injuries all arrived to HUCSH within 24 h, irrespective of mode of transport. The majority of ambulances (65%) were utilised for patients with a mild KTS, with only 25% of ambulances transporting moderate and severe injuries. All severe injuries transported by ambulance were patients referred from other centres.

Once in hospital, 21% of patients were not triaged on admission; the majority of these (54%) were direct admissions. Sixty-nine per cent of patients stayed in ED for more than 24 h (Table 3).

**Table 3:** 24 h outcomes

*Patient 24 h outcomes*	*Total*
Kept in ED > 24 h	1327 (69%)
Admitted	356 (19%)
Medical discharge	135 (7%)
Self-discharge	45 (2%)
Transferred out	37 (2%)
Other	4 (< 1%)
*Deaths*	*Total*
< 24 h	15 (1%)
> 24 h	11 (< 1%)

There were a total of 376 patients (20% of all trauma patients) who presented with an open fracture—36% of all fracture/dislocations. The average age for these patients was 28 years, and the majority (81%) were male. Seventy-six per cent of open fractures had low KTS and a large proportion (67%) of open fracture patients were kept in ED for > 24 h (Table 4).

**Table 4:** Open fractures

	Total	Arrival < 24 h	Arrival > 24 h
*Open fractures*			
Total	577 (30%)	385 (67%)	192 (33%)
Male	469 (81%)		
Female	108 (19%)		
*Origin of open fractures*			
Referred	477 (83%)	305 (64%)	172 (36%)
Direct	99 (17%)	79 (80%)	20 (20%)
*Open fracture versus KTS*			
Average KTS	14.7		
Median (SD) KTS	15 (SD 1.08)		
Mild KTS	437 (76%)		
Moderate KTS	58 (10%)		
Severe KTS	1 (< 1%)		
No data	81 (14%)		
*Open fracture 24 h outcomes*			
Admitted	164 (28%)		
Direct to theatre	1 (< 1%)		
Kept in ED > 24 h	370 (64%)		
Discharged	38 (7%)		
Died	1 (< 1%)		
Transferred	5 (< 1%)		

## Discussion

Our 12-month data highlight ambulance utilisation as a potential area for improvement. Ninety per cent of ambulances are used to transfer patients from other centres with 65% of these patients having a mild KTS. Only 25% of ambulance transfers were for moderate or severe injuries (Table 2). We found that all ambulances utilised for severely injured patients were transfers from other centres. Not only does this highlight the absence of a ‘scoop service’ in the local area, but also that ambulance allocation could be improved to ensure those with moderate and severe injuries are transferred promptly to an appropriate health facility.

Our data provide evidence of a degree of injury severity prioritisation, although improvement is needed. A standardised triage tool has not been utilised across the region, but our data do show that the more severe injuries all arrived to HUCSH within 24 h, irrespective of mode of transport. Seventy-seven per cent of all trauma patients at HUCSH were referred from other centres, a large proportion of these patients—69%—had a mild KTS. Only 17% of patients referred had a moderate KTS and < 1% a severe KTS. Such high numbers of low severity referrals highlight the need for a more robust referral criteria to avoid overwhelming the tertiary centre—blocking valuable beds for the most severe injuries, and negatively contributing to patient flow and care in the emergency department.

The regional use of effective triage tools for both life- and limb-threatening injuries could significantly improve severity prioritisation. The effectiveness of the KTS as a point of care triage tool in LMIC trauma patients has previously been questioned [10]; our data suggest that a secondary triage tool for limb-threatening injuries may be beneficial. We found that 76% of open fractures had a low KTS (Table 4), highlighting that current trauma scoring tools fail to take into account the impact of open fractures and the need for a secondary triage tool for limb-threatening injuries.

With regard to the management of open fractures, our data show that a large proportion (67%) of these patients remained in the emergency department for more than 24 h (Table 4). Although these patients received IV antibiotics in the department, this raises concerns regarding whether or not time to debridement exceeded 24 h. Open fractures are complex injuries that can be associated with debilitating complications such as neurovascular compromise, infection and long-term impact on a patient’s ability to work [12, 13]. Early recognition and standardised treatment of these conditions is vital to avoid both short- and long-term sequelae.

Our data also highlighted problems with patient flow, with 69% of patients remaining in the emergency department for more than 24 h (Table 3). Initiating tighter referral criteria can contribute to a reduction in overall numbers, improved initial assessment and management of trauma patients to ensure patients with mild injuries are treated and discharged promptly with outpatient care. Other contributing factors are the cost-sharing nature of the Ethiopian health system and blood product shortages which impact on all 3 delays in the access to healthcare model, time to theatre and patient flow.

In comparison with trauma registry data from other sub-Saharan LMIC’s, we have found that although we can draw similarities, there are also many differences to consider. In terms of similarities, the majority—young male demographic is consistent. Problems with incomplete recording of vital signs and incomplete or poor-quality data are also a common theme [14]. Notable differences between trauma registries were the mechanism of injury, ambulance utilisation and 24-h outcomes. Trauma registries from Malawi and South Africa have published data showing ‘Assault’ as the most common mechanism of injury [14, 15], in comparison with the Hawassa trauma registry which shows ‘road traffic collisions’ account for 53% of injuries, and ‘assaults’ only accounting for 8%. The data published in 2019 from the trauma registry at the Queen Elizabeth Central Hospital (QECH) in Blantyre—Malawi, demonstrated a very different distribution of mode of transport—with only 3.4% of patients using an ambulance in comparison with the Hawassa data where ambulances were used in 39% of trauma patients [14]. The QECH data also showed that only 14.3% of trauma patients were admitted to wards—inferring a high proportion are treated as outpatients; this is a significant difference to the Hawassa data which showed that 28% of trauma patients had been admitted to a ward within 24 h. Whilst QECH and HUCSH are both referral hospitals, it is important to note that QECH serves a large city of 800,000 people as well as a referral population of approximately 8 million; in comparison with HUCSH, a hospital which serves a smaller town of 320,000 but a huge referral population of 21 million. This demonstrates the need for focused country—and possibly, region—specific data to accurately guide resource allocation, further research and standardisation of care. Differences highlight that trends from one LMIC’s trauma registry should not be assumed to apply to other LMICs in a ‘one size fits all’ approach.

Moving forward, the first year of data from the Hawassa trauma registry has highlighted key areas for improvement. The introduction and dissemination of a more robust referral criteria and pathway can contribute to improved ambulance utilisation as well as reducing the numbers of inappropriate referrals and subsequent bed blocking in the Hawassa emergency department. A referral criterion utilising a validated trauma scoring tool and incorporating the presence of open fractures may help to ensure the right patients are appropriately referred in a timely fashion. Highlighting severely injured patients and those with limb-threatening injuries can also serve as a prompt to initiate appropriate trauma resuscitation and management, irrespective of whether the patient is for transfer to the tertiary centre. Appropriate utilisation of ambulance services for moderately and severely injured patients may allow for the introduction of a local ‘scoop’ ambulance trauma service.

Similarly to those outlined in other LMIC trauma registry papers [14, 15], there were various difficulties faced during the data collection process. Variable or incomplete data collection meant that the quality of data was not as high as it could have been. Blank box data forms also provided opportunity for transcription errors and an element of researcher interpretation. High clinician turnover and gaps in data collection training could have also negatively contributed to data quality. Moreover, the recorded deaths were limited to those occurring in-hospital and there were no data collected regarding the numbers of patients requiring surgery; as a result of this, it is likely that many trauma deaths have not been included in our figures.

Not extending data collection to the district general hospitals meant that data relating to care received prior to arrival in Hawassa Hospital were missing or incomplete. Patient outcomes were only documented for the first 24 h which could have skewed our mortality data and outcome interpretation.

Incomplete measurement of vital signs meant 21% of patients were not triaged on admission. Ensuring this happens, will allow prompt referral and resuscitation of patients. Although lack of appropriate equipment—such as, appropriately sized blood pressure cuffs and pulse oximeters—contributes to this, future work to improve the registry needs to place emphasis on educating clinicians, emergency nurses and other practitioners with regard to triaging, clear documentation of clinical observations and the interpretation of trends in vital signs for trauma patients.

Building on previous discussions between the trauma network and district general hospitals will be needed to improve training in basic trauma care and improving communication between centres. Communication regarding referrals and general advice via WhatsApp or, preferably, a ‘trauma coordinator’ role may be beneficial in reducing the numbers of inappropriate and unnecessary referrals and ensuring district general hospitals have the expertise and support to treat less severe injuries locally.

The introduction of guidelines for the management of open fractures has been implemented in Malawi [16] to standardise care and is something that could be explored in other LMICs, including Ethiopia. Time to the operating theatre needs to be improved and focused data collection on the treatment of open fractures is needed. Good communication and teaching of guidelines to healthcare workers in district general hospitals and referral centres is key to improving the outcomes for these patients.

Additional data to collect in the future relate to the use of blood products, trauma resuscitation and care received at presenting medical facility. The data collection window will be increased to > 24 h, covering entire length of stay, to provide a better picture of patient pathways and provide more reliable mortality data. Comparative data to assess the impact of conflict in the north of the country will include trauma volume, delayed presentations and numbers of severe injuries—allowing assessment of the resilience of the current trauma system.

We intend to collect more detailed fracture/dislocation data to further analyse this category. Data regarding fracture location and type of fracture were collected in a free text box but were missing, incomplete or documented in a way that could not be reliably interpreted. Future data will be collected via a clear checkbox criteria and anatomical location of fractures will be included. Focused data collection on the treatment of open fractures is needed and will include time to first antibiotic dose, tetanus cover and time to first debridement—this will allow a comparison to open fracture guidelines utilised around the world.

In conclusion, given the current political climate in the north of the country, having a resilient and efficient trauma network is essential. We have shown that a ‘one size fits all approach’ should not be adopted for LMIC’s as trends vary between regions and countries. Improvements are needed in referral pathways, the management of open fractures, ambulance utilisation and the use of appropriate triaging tools to facilitate initial basic trauma care and appropriate, timely referrals. We hope this study will aid future development of existing trauma care systems and injury prevention framework in Hawassa and lead to a reduction in numbers of deaths and severely disabled persons.

## Supplementary Information

Below is the link to the electronic supplementary material.

Supplementary file1 (DOCX 22 KB)
