# Supplementary material for: Use of a trauma registry to drive improvement in the regional trauma network systems in Hawassa, Ethiopia
Source: Eur J Orthop Surg Traumatol. 2022 Oct 29;33(3):541–6. doi: 10.1007/s00590-022-03410-z (PMC9616696; doi:10.1007/s00590-022-03410-z)
Supplement: Supplementary file 1 — Supplementary file1 (DOCX 22 KB) [file 590_2022_3410_MOESM1_ESM.docx]

**Supplementary Data**

**Table 1: Time to Hospital vs KTS score + Mode of Transport:**

|  | **Ambulance** | **Public Transport** | **Private Transport** | **Other/ No data** |
| --- | --- | --- | --- | --- |
| **Total** | 747 (39%) | 777 (40%) | 385 (20%) | 10 (<1%) |
|  | **Time to Hospital (hours)**  **Ambulance** | **Public Transport** | **Private Transport** | **Other/No Data** |
| **Mild KTS (1321)** | 483  (37%) | 552 (42%) | 278 (21%) | - |
| Median (SD) | 10 (SD 134) | 18 (SD 448) | 8 (SD 160) | - |
| Range | 1-2160 | 1-8640 | 1-1440 | - |
| <24h | 72% | 52% | 71% | - |
| >24h | 28% | 48% | 29% | - |
| **Moderate KTS** | 181 (57%) | 78 (25%) | 55 (17%) | - |
| Median (SD) | 8 (SD 224) | 12 (SD 77) | 7 (SD 35) | - |
| Range | 1-2880 | 1-408 | 1-216 | - |
| <24h | 75% | 60% | 76% | - |
| >24h | 25% | 40% | 24% | - |
| **Severe KTS** | 3 (33%) | 4 (44%) | 2 (22%) | - |
| Median (SD) | 11 (SD 10) | 10.5 (SD 9.6) | 5 (SD 1.4) | - |
| Range | 1-20 | 1-19 | 4-6 | - |
| <24h | 100% | 100% | 100% | - |
| >24h | - | - | - |  |
| **No data** | 80 (30%) | 141 (51%) | 50 (19%) | - |

**Table 2. Injury Type:**

| **Injury Type** | **Primary injury** | **Total (including secondary injuries)** |
| --- | --- | --- |
| **Fracture/ Dislocation** | 941 (49%) | 1043 (54%) |
| **Head Injury** | 713 (37%) | 796 (42%) |
| **Soft Tissue Injury** | 138 (7%) | 178 (9%) |
| **Internal Organ Injury** | 69 (4%) | 137 (7%) |
| **Spinal Injury** | 45 (2%) | 61 (3%) |
| **Other** | 11 (<1%) | 12 (<1%) |
| **No data** | 2 (<1%) | 2 |

**Table 3. Deaths:**

| **Total Deaths** | 26 (1%) |  |  |
| --- | --- | --- | --- |
| **Origin** |  |  |  |
| Direct admission | 6 (23 %) |  |  |
| Ambulance | 2 |  |  |
| Public Transport | 2 |  |  |
| Private Transport | 2 |  |  |
| Referred | 20 (77%) |  |  |
| Ambulance | 13 |  |  |
| Public Transport | 3 |  |  |
| Private Transport | 4 |  |  |
|  |  |  |  |
|  |  | **Time to Hawassa (h)** |  |
| **KTS** |  | **Median (SD)** | **Range** |
| Mild KTS | 5 (19%) | 8 (SD 201) | 4-456 |
| Referred | 5 |  |  |
| Direct | 0 |  |  |
| Moderate KTS | 17 (65%) | 7 (SD 59) | 3-216 |
| Referred | 13 |  |  |
| Direct | 4 |  |  |
| Severe KTS | 2 (8%) | 5 (SD 1.4) | 4-6 |
| Referred | 1 |  |  |
| Direct | 1 |  |  |
| No data | 2 (8%) | 7.5 (SD 0.7) | 7-8 |
| Referred | 1 |  |  |
| Direct | 1 |  |  |
|  |  |  |  |
| **Injury type**  **(primary + secondary injuries)** |  |  |  |
| HI | 18 (69%) |  |  |
| Fracture/ Dislocation | 8 (30%) |  |  |
| Spinal Injury | 1 (<1%) |  |  |
| Internal Organ Injury | 4 (15%) |  |  |
| Soft Tissue Injury | 1 (<1%) |  |  |

**Table 4. Triage**

| **Triage on admission** |  |
| --- | --- |
| Green | 181 (9%) |
| Yellow | 883 (46%) |
| Orange | 298 (16%) |
| Red | 161 (8%) |
| No Triage | 396 (21%) |
| **Origin of non-triaged patients** |  |
| HUCSH | 213 (54%) |
| Referred | 183 (46%) |
|  |  |
